# Engendered nanoparticles for treatment of brain tumors

**DOI:** 10.32604/or.2024.053069

**Published:** 2024-12-20

**Authors:** SOROUSH SOLEYMANI, MOHAMMAD DOROUDIAN, MAHDIEH SOEZI, ALI BELADI, KIARASH ASGARI, ASO MOBARAKSHAHI, ARYANA AGHAEIPOUR, RONAN MACLOUGHLIN

**Affiliations:** 1 Department of Cell and Molecular Biology, School of Biological Sciences, University of Leicester, Leicester, LE1 7RH, UK; 2 Department of Cell and Molecular Biology, Faculty of Biological Sciences, Kharazmi University, Tehran, 15719-14911, Iran; 3 Cancer Epidemiology Research Center, Aja University of Medical Sciences, Tehran, 14117-18541, Iran; 4 Medical Biotechnology Research Center, Aja University of Medical Sciences, Tehran, 14117-18541, Iran; 5 Department of Life Sciences, Faculty of Natural Sciences, Imperial College London, London, SW7 2AZ, UK; 6 School of Pharmacy and Biomolecular Sciences, Royal College of Surgeons in Ireland (RCSI), Dublin, D02 YN77, Ireland; 7 Research and Development, Science and Emerging Technologies, Aerogen Ltd., Galway Business Park, Galway, H91 HE94, Ireland; 8 School of Pharmacy and Pharmaceutical Sciences, Trinity College, Dublin, D02 PN40, Ireland

**Keywords:** Nanoparticles, Smart nanoparticles, Stimuli responsive nanoparticles, Targeted nanoparticles, Blood-brain barrier (BBB), Brain cancer

## Abstract

Brain metastasis and primary glioblastoma multiforme represent the most common and lethal malignant brain tumors. Its median survival time is typically less than a year after diagnosis. One of the major challenges in treating these cancers is the efficiency of the transport of drugs to the central nervous system. The blood-brain barrier is cooperating with advanced stages of malignancy. The blood-brain barrier poses a significant challenge to delivering systemic medications to brain tumors. Nanodrug delivery systems have emerged as promising tools for effectively crossing this barrier. Additionally, the development of smart nanoparticles brings new hope for cancer diagnosis and treatment. These nanoparticles improve drug delivery efficiency, allowing for the creation of targeted and stimuli-responsive delivery methods. This review highlights recent advancements in nanoparticle and smart nanoparticle technologies for brain cancer treatment, exploring the range of nanoparticles under development, their applications, targeting strategies, and the latest progress in enhancing transport across the blood-brain barrier. It also addresses the ongoing challenges and potential benefits of these innovative approaches.

## Introduction

The brain organizes most of the body’s functions and processes. The brain controls breathing movements and digestion through sensory processing [1]. The cerebellum covers approximately 10 percent of the brain, and it accompanies the right and left hemispheres. They are responsible for performing higher sensory and motor functions and controlling movements [1,2]. The brain stem conducts a variety of automatic tasks. Furthermore, the appropriate propagation of behavioral processes is linked to the brain’s four lobes [1,3]. Brain diseases can cause serious damage to an individual’s body and life span [1]. Brain cancer is considered one of the most fatal diseases. The BBB is a significant limitation to treatment efficacy in brain cancer [4–6]. The BBB and strategies for minimizing its effect shall be discussed in detail throughout this review. Based on the information provided by the National Brain Tumor Society, 78,000 individuals are diagnosed with malignant brain tumors yearly in the United States [7]. The abnormal static and metastatic proliferation of cells can lead to benign and malignant tumors. Increased intracranial pressure, headaches, vomiting, altered consciousness, and seizures are among the symptoms of brain tumors. Brain tumors are typically defined based on their size and location within the brain. Glial cells are the most prevalent site of tumor invasion. Low-grade tumors are classed as Grades I and II [8,9], while higher-grade tumors are classified as Grades III and IV [10].

Among various cancer treatments, targeted therapies have shown promising results compared to traditional methods like chemotherapy. Conventional therapies often cause several undesired side effects, which can cause a tendency to prescribe lower doses than necessary for effective treatment [11]. Recent studies, including clinical trials outlined in Table 1, have expanded the scope of nanoparticle (NP) applications beyond traditional roles in drug delivery, imaging, and gene therapy. These studies explore the multifaceted potential of NPs, delving into advanced therapeutic strategies and diagnostic enhancements [12–14].

**Table 1:** Examples of clinical trials employing nanoparticle drugs for gliomas (https://clinicaltrials.gov/)

Therapeutic agent	Diseases	Phase	Clinical trial
Pegylated Liposomal Doxorubicine + Temozolomide	Glioblastoma and diffuse intristic pontine glioma	Completed	NCT00944801
ABI-009 (nab-rapamycin)	High-grade glioma; Newly diagnosed glioblastoma	II	NCT03463265
9-ING-41	Glioblastoma	II	NTC03678883
SGT-53	Recurrent glioblastoma II	II	NTC02340156
Doxorubicin	Glioblastoma and diffuse intristic pontine glioma	II	NCT02758366
NL CPT-11 (Nanoliposomal CPT-11)	High-grade glioma	I completed	NCT00734682
2B3-101	Malignant glioma or solid tumors and brain metastases	I/II	NCT01386580
Ferumoxytol	High-grade glioma	I	NCT00769093
Myocet	Refractory or relapsed malignant glioma in children/adolescent	I	NCT02861222
NU-0129	Glioblastoma or gliosarcoma	I	NCT03020017
RNL (rhenium nanoliposomes)	Glioblastoma	I	NCT01906385

**Figure 1:**
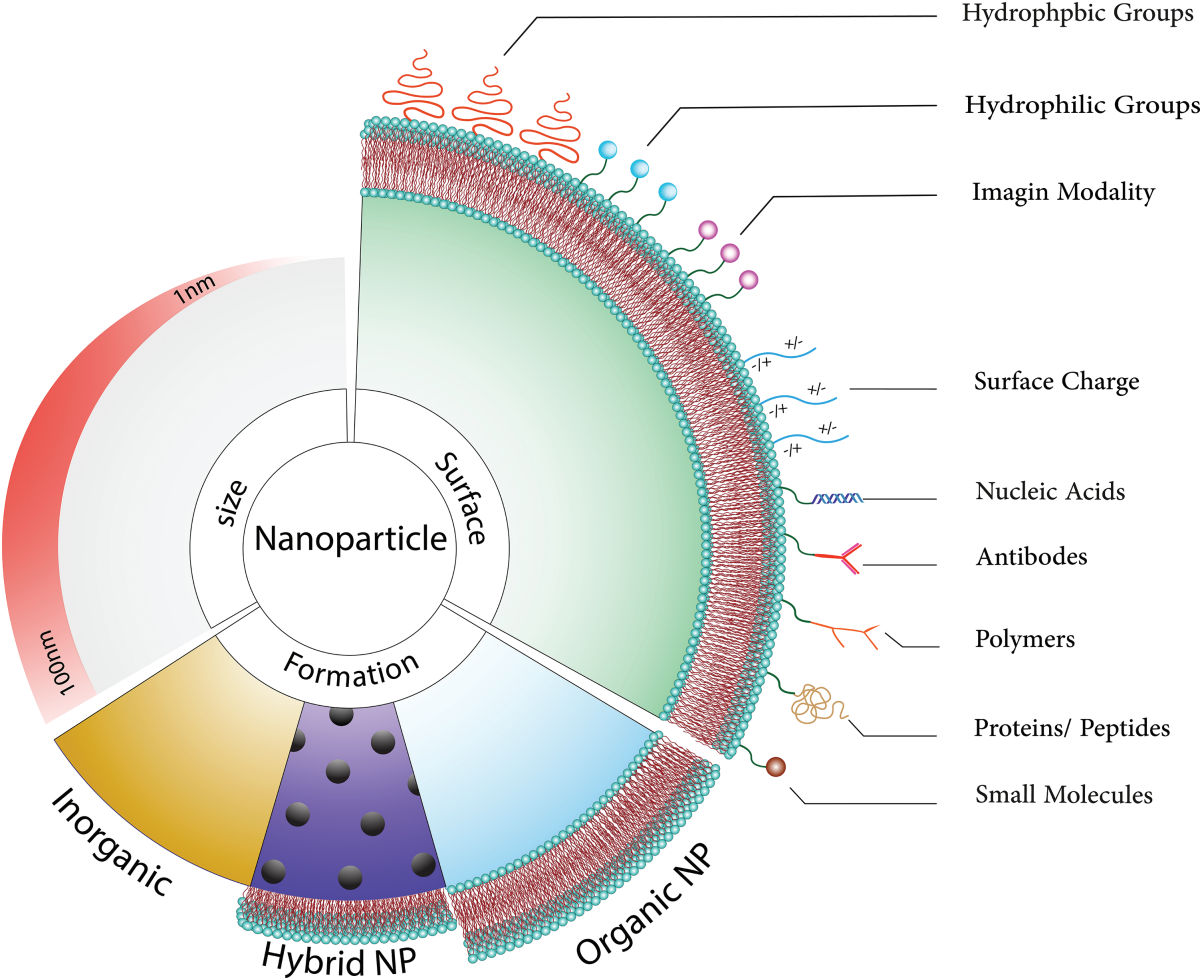
Nanoparticles can be thought of as ingenious couriers in the medical field, possessing an exceptional ability to assist medicines in reaching the brain by bypassing the blood-brain barrier. These diminutive heroes not only transport therapeutic agents but also provide protection and precise release at the intended site. Remarkably, these nanoparticles can be customized to execute diverse tasks, such as reducing their size to less than 100 nm, enabling them to effortlessly traverse biological barriers like agile acrobats. Furthermore, we have the innovative option to integrate different types of nanoparticles to create hybrids with augmented capabilities [18]. Organic nanoparticles, recognized for their compatibility with the human body and minimized harm, present immense potential for various applications [19,20]. By modifying the molecules on their surface, we can impart these nanoparticles with unique abilities, including electrical charges or the aptitude to target specific areas. Essentially, comprehending these nanoparticle experts and refining their design is akin to possessing a Swiss Army knife for drug delivery, presenting limitless possibilities for more precise and efficacious medical treatments [21–23].

**Figure 2:**
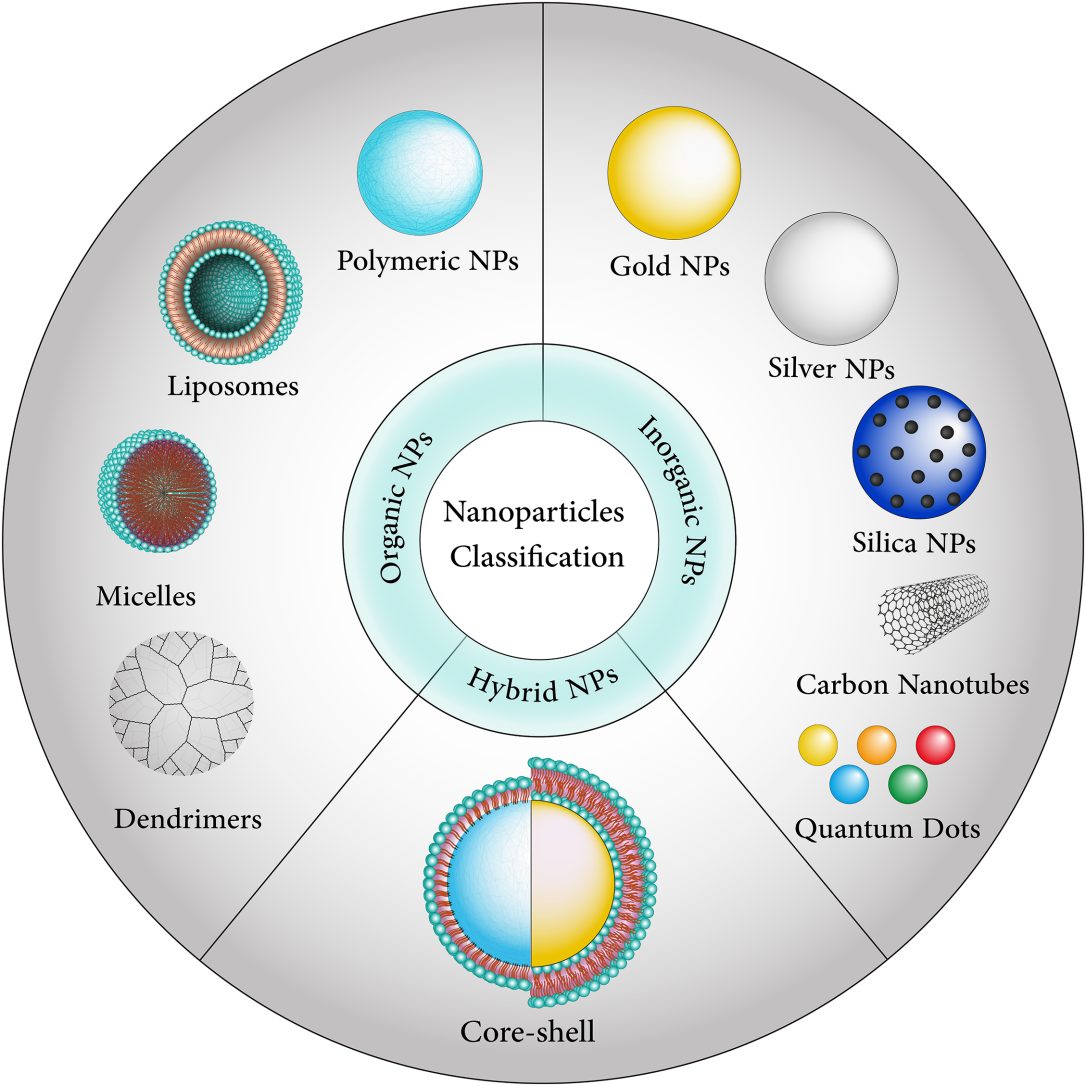
Nanoparticles are divided into different categories, including organic, inorganic, and hybrid-based.

Extensive research has been conducted on nanoparticles due to their remarkable potential to traverse the BBB [15,16] (Figs. 1 and 2). In this review, we detail the current state of the art in active and passive targeting nanoparticle delivery systems. In the active targeting of cancer cells with high affinity and precision, the nanoparticles can be coupled with tumor-targeting moieties such as particular ligands, monoclonal antibodies, and carbohydrates [17]. We also provide an overview of brain cancer treatments using nanoparticles, covering the diversity of nanoparticles, some drug transportation mechanisms, and the most common issues encountered by these various high-potential drug delivery systems.

### The blood-brain barrier as an obstacle to brain tumor drug delivery

The BBB is a barrier that separates the body’s circulatory system from the central nervous system (CNS) [24–26]. It is a diffusion barrier that prevents pathogens, neurotoxic plasma components, blood cells, therapeutic substances, and other chemicals from entering the brain, thereby maintaining brain homeostasis [27–30].

Endothelial cells (ECs), pericytes, astrocytes, tight junctions (TJs), neurons, and the basement membrane work in concert to ensure that the brain capillaries are virtually pore-free, thereby maintaining the integrity of the BBB and preventing the transport of small molecules and proteins. The BBB’s permeability is influenced by physiochemical characteristics such as molecular weight, charge, lipid solubility, surface activity, and the relative size of the molecules. These molecules can pass through paracellular conduits (between adjacent cells) or transcellular channels (through cells) of the BBB.

In the paracellular pathway, ions and solutes migrate across the BBB via passive diffusion, exploiting concentration gradients. Additionally, small lipophilic molecules like carbon dioxide can pass through the BBB via the transcellular pathway through passive diffusion [29,31,32]. Physiological factors that influence BBB permeability include efflux transporters (i.e., P-glycoprotein (P-GP)), enzymatic activity, and plasma protein concentration [33–36].

The breakdown of the BBB in disease conditions results in the leakage of toxic blood components into the CNS, cellular infiltration, and abnormal transport resulting in neurological impairments [27,37].

### Strategies for overcoming the blood-brain barrier

The BBB presents a significant obstacle in the delivery of drugs to the brain, permitting only small, lipophilic molecules to pass through naturally. Complicating this challenge, the BBB is further protected by drug efflux transporters, predominantly from the ATP-binding cassette (ABC) superfamily, which frequently expels drugs trying to cross [38]. Overcoming this obstacle is essential for the treatment of brain cancer, given its intricacy, side effects, and the BBB’s resistance to most macromolecules [33,39].

To navigate this intricate barrier, nanomedicine has emerged as a promising solution [40–42]. Diverse inorganic, organic, and natural nanomaterials, enhanced with BBB-targeting ligands and cell-penetrating peptides (CPPs), have been engineered to facilitate drug delivery and theranostic approaches for brain tumors [43,44]. This transformative nanomedicine is poised to revolutionize brain cancer treatment by creating multifunctional BBB-crossing nanoparticles with remarkable properties like magnetic, optical, thermal, and tumor microenvironment-responsive capabilities [45,46].

Ultimately, the physiological role of the blood-brain barrier remains the primary culprit behind complications in current drug delivery strategies, emphasizing the critical need for innovative approaches to brain cancer treatment [47–50].

Nanoparticles offer a promising strategy for delivering therapeutic agents across the BBB [51]. The mechanism by which nanoparticles cross the BBB is multifaceted and involves several strategies. One of the primary methods is receptor-mediated transcytosis where nanoparticles are engineered to bind to specific receptors on the BBB, triggering a process that transports them across the barrier. Another mechanism is adsorptive-mediated transcytosis, which relies on the electrostatic interaction between the positively charged nanoparticles and the negatively charged cell membranes, facilitating their entry into the brain [52]. Nanoparticles can also be designed to temporarily disrupt the BBB, allowing them to pass through. This can be achieved by adjusting the nanoparticle’s surface properties or by using external forces like magnetic fields if the nanoparticles are magnetic [53]. Additionally, cell-penetrating peptides (CPPs) attached to nanoparticles can help them penetrate the BBB by inducing endocytosis [53].

Furthermore, nanoparticles can exploit natural transport mechanisms such as carrier-mediated transcytosis for substances like glucose or amino acids, by mimicking these molecules [53]. The intranasal route is another approach, offering a direct connection to the brain via the olfactory and trigeminal nerves, bypassing the BBB entirely. These mechanisms are under continuous research and development, aiming to enhance the delivery of therapeutics to the brain for the treatment of various central nervous system disorders. The ultimate goal is to achieve efficient and targeted delivery without compromising the integrity of the BBB or causing adverse effects [53].

### The role of smart nanoparticles in the treatment of brain tumors

In the realm of advanced medical science, smart nanoparticles emerge as remarkable tools to overcome the formidable barrier known as the BBB and offer hope in treating brain tumors. Smart nanoparticles are devised to ferry drugs and therapeutic genes (in this case, anticancer drugs) into virulent cells without any lethal effect on normal cells [54,55]. The critical facet of tumor treatment is delivering as many drugs as possible to the targeted cells [56,57]. These nanocarriers, including lipid materials, polymers, nanocrystals, and inorganic nanomaterials, possess distinctive structural attributes that enable them to effectively traverse the BBB and precisely target elusive glioma cells [58]. Among the promising candidates for drug delivery in brain diseases, gold nanoparticles (AuNPs) are highly notable due to their adjustable size, optical characteristics, surface adaptability, and strong biocompatibility [59]. Meanwhile, clinical feasibility has introduced strategies involving liposomes, extracellular vesicles, and biomimicry, with liposomes emerging as particularly effective methods for enhancing drug delivery to the brain [34]. Numerous anticancer medications exhibit hydrophobic properties, which allows them to integrate seamlessly into the liposomal lipid bilayer. This characteristic facilitates their transport to other hydrophobic regions within the bloodstream [56,60]. A rat model study manifests that Liposomes with firm bilayers provide a higher transfer rate than liposomes with pliable bilayers. Moreover, it showed that PEGylated Liposomes are capable of releasing a hydrophobic drug at a higher speed rate in comparison to the control [56,61]. Another intriguing method reveals itself with magnetic nanoparticles (MNPs), coated with gold and linked to polyethylene glycol (PEG), exhibiting the potential to efficiently navigate the BBB under the influence of an external static magnetic field (SMF) [62]. Furthermore, nanoparticle customization through the integration of anti-transferrin receptor antibodies emerges as an exciting approach, allowing for precise targeting of brain endothelial cells (BECs) and seamless passage through the BBB, thus illuminating a promising path for the future delivery of biologics to the brain [63].

Smart delivery systems have been fabricated to define different stimuli. These external factors are ultrasound, radiofrequency, light, and temperature [56]. Ultrasound, a pressure wave, has excellent spatial and temporal resolution. The energy wasted during ultrasound interaction with tissues can cause heating and non-thermal mechanical effects. In drug delivery, a complex of ultrasound and microbubbles is being utilized. Microbubbles are capable of producing a diversity of mechanical forces when they are confronting ultrasound. By treating a particular site with concentrated ultrasound, sonoporation can be employed to augment the extravasation of drugs from blood to interstitial space [64,65]. More smart drug delivery methods using nanoparticles have shown remarkable improvements in cancer therapeutics, including active and passive targeted delivery systems [66].

## Targeted Nanoparticles

Targeted nanoparticles represent precision-guided missiles within the sphere of drug delivery, exhibiting considerable potential to enhance the delivery and efficacy of therapies [67]. To address the obstacles presented by glioblastoma multiforme (GBM), scientists have designed polymeric nanoparticles that respond to Chlorotoxin, honing in on cancer cells and prompting their selective elimination [68]. Through the creation of a versatile nanoparticle platform, they have achieved an impressive degree of precision in targeting tumors, while concurrently minimizing any unintended adverse effects [69]. Additionally, in the realm of proteomics, modified nanoparticles have been employed to selectively retrieve peptides and proteins, creating high-throughput platforms that promote the advancement of our comprehension of intricate biological systems [70].

Zhu et al. developed an investigation around the creation of ANG-LP-MSN-PTX, a variant of mesoporous silica nanoparticles coated with lipids and modified with angiopep-2, for the aim of treating glioma, a deadly brain tumor, using targeted therapy. LRP1 (low-density lipoprotein receptor-related protein 1), a receptor protein that is abundant on the surface of both BBB and glioma cells, is planned to transport Paclitaxel (PTX) across the BBB using Nanoparticles. ANG-LP-MSN-PTX exhibited uniform hydrodynamic size, elevated drug loading capacity, and sustained release. Subsequent *in vitro* experiments revealed heightened targeting efficiency and increased apoptosis of C6 glioma cells. Pharmacokinetic analyses further demonstrated superior targeting efficiency of ANG-LP-MSN-PTX relative to LP-MSN-PTX. LP-MSN-PTX is a widely used chemotherapeutic agent, to target cells. This system typically involves loading PTX into mesoporous silica nanoparticles (MSNs) and then coating them with a lipid layer (LP), which helps to improve the stability and biocompatibility of the nanoparticles. Notably, *in vivo* experiments conducted on intracranial C6 glioma-bearing rats evinced increased therapeutic efficacy and prolonged survival time with ANG-LP-MSN-PTX. Ultimately, ANG-LP-MSN-PTX represents a promising targeted delivery system for the treatment of glioma [71].

Emerging medical approaches such as gene therapies, immunotherapies, targeted treatments, small molecules, and antibodies have been extensively studied to improve the survival rates of patients with glioblastoma multiforme (GBM) [45] Among these, the first approach, Nucleic Acid Therapeutics, provides precise control over gene expression. It utilizes DNA and mRNA to express specific genes that combat GBM, enhancing apoptosis in GBM cells or making them more receptive to chemotherapy [72]. Additionally, siRNA and miRNA selectively silence genes that contribute to GBM progression, angiogenesis, and immune suppression [73,74]. The versatility of nucleic acids also allows for customized treatments targeting unique genetic factors within individual tumors [75,76]. Furthermore, CRISPR-Cas9 technology enables gene editing to knock out oncogenes specific to GBM [77].

The second approach is Immunotherapy, which harnesses the immune system against GBM. Although once considered immune-privileged, recent studies have shown immune activity in the central nervous system [78]. Despite GBM creating a highly immunosuppressive microenvironment, immunotherapies such as checkpoint inhibitors and cancer vaccines aim to overcome these challenges [79,80]. Checkpoint inhibitors target immune checkpoints like PD-1, and personalized cancer vaccines based on neoantigens have shown promise. Additionally, CAR T-cell therapy introduces engineered T cells specific to GBM antigens into the patient [81]. However, resistance mechanisms and the need for combination therapies pose significant challenges in immunotherapy [81,82].

The third approach, Suicide Gene Therapy, involves reprogramming cancer cells to undergo targeted apoptosis upon exposure to a prodrug. Herpes simplex virus thymidine kinase (HSV-tk) is one such gene used, where HSV-tk-expressing cancer cells convert a non-toxic prodrug like ganciclovir into a toxic substance, leading to cell death [83,84]. However, mixed results in clinical trials have highlighted the need for improved gene delivery methods and higher transfection efficiency [85,86].

The fourth approach involves Small Molecules and Antibodies. Genetic and epigenetic screening has identified molecular targets in GBM, such as often overexpressed or mutated tyrosine kinase receptors EGFR and PDGFR, along with targets like VEGF in the tumor microenvironment [87]. Small molecule inhibitors can block the activity of these receptors, disrupting essential signaling pathways for GBM growth. Monoclonal antibodies, meanwhile, can neutralize factors like VEGF [88]. Despite the potential of these targeted therapies, GBM’s heterogeneity and the development of resistance mechanisms present challenges. These therapies may be more effective when used in combination with other treatments [45,89].

Each of these approaches holds potential in the fight against GBM but also comes with unique sets of challenges that require further research and development for more effective treatments.

## Stimuli Responsive Nanoparticles

Stimuli Responsive Nanoparticles are advanced materials designed to react to specific triggers such as pH and temperature, enabling controlled drug delivery and release. These smart nanosystems adapt their behavior in response to the body’s microenvironment or external stimuli, offering precision in treatments. Stimuli-responsive nanoparticles possessing intelligent features have surfaced as a promising technique for targeted drug delivery in biomedical domains [90]. These nanoparticles do not seem to be customizable to interact with any specific stimuli that are present in the tumor microenvironment, including high glutathione concentration, acidity, reactive oxygen species (ROS), hypoxia, and over-expressed enzymes [91]. pH and redox, nanoparticles that respond to endogenous stimuli, and polymeric nanoparticles that can be transformed into systems that are sensitive to stimuli. These nanoparticles can sustain stability in physiological conditions and discharge drugs exclusively in tumor tissues, providing improved therapeutic efficacy and better biosafety [90,92–94].

With the advancement of nanomedicine technology, stimuli-responsive nanocarriers have become increasingly prominent in antitumor therapy. The tumor microenvironment (TME) is distinguished by unique characteristics, such as sourness, raised glutathione (GSH) concentrations, lack of oxygen, hyperactive enzymes, and ample reactive oxygen species (ROS), all of which are intricately tied to tumor development. Interestingly, these TME characteristics can be leveraged for intelligent drug delivery systems that target tumor tissues. Stimuli-responsive nanoparticles (srNPs) exhibit stability in physiological conditions but can rapidly release drugs upon specific triggers, extending circulation and enhancing cancer cell uptake, ultimately achieving superior therapeutic efficacy and enhanced safety [95].

This study delves into the challenges and future directions in the development of stimulus-responsive nanoparticles (srNPs), shedding light on their potential clinical implications. Nanomedicine has notably transformed drug delivery to tumors, leveraging unique molecular characteristics. Despite the enhancements nano drug delivery systems (NDDS) have brought to traditional chemotherapy, optimizing drug bioavailability within tumor tissues remains critical, especially in terms of cellular uptake and intracellular release. srNPs, designed with an intricate understanding of the disparities between normal and cancerous tissues, render this review an invaluable guide to the progress in srNPs for cancer treatment. The review briefly covers the endogenous stimuli of the tumor microenvironment (TME)—such as low pH, elevated glutathione (GSH) levels, overexpressed enzymes, excessive reactive oxygen species (ROS), and hypoxia—summarizes srNPs’ therapeutic applications, and encourages further research to hasten their clinical integration. Additionally, Table 2 is introduced to discuss the properties of the TME in detail [92,96]. These characteristics collectively contribute to the complex and challenging nature of the tumor microenvironment, impacting cancer progression and therapeutic strategies.

**Table 2:** Tumor microenvironment (TME) properties [92]

Characteristic	Description	Significance in TME
Acidity	TME is more acidic (pH 6.5) compared to normal cells (pH 7.4). Acidic TME promotes tumor growth, metastasis, and drug resistance.	Acidic conditions favor tumor progression and MDR.
High GSH concentration	Tumor tissues have 10 times higher GSH levels than normal tissues. GSH is crucial for detoxification and immune functions.	Abnormal GSH levels are linked to various diseases.
Hypoxia	TME experiences oxygen deficiency due to irregular blood vessel structures. Hypoxia aids tumor invasion, metastasis, and drug resistance.	Hypoxia-induced factors (HIFs) enhance tumor growth.
Overexpressed enzymes	Tumor tissues exhibit overexpression of specific enzymes in the TME, such as MMPs. Enzyme-targeted srNPs can release drugs in the TME.	Abnormal enzyme activity is the basis for many diseases.
Excessive ROS	Tumor cells have significantly higher ROS levels (10^−4^ M) compared to normal tissues (2 × 10^−8^ M). ROS play roles in tumorigenesis.	Excessive ROS can lead to tissue damage and diseases.

### Passive and active targeting drug delivery systems

**Figure 3:**
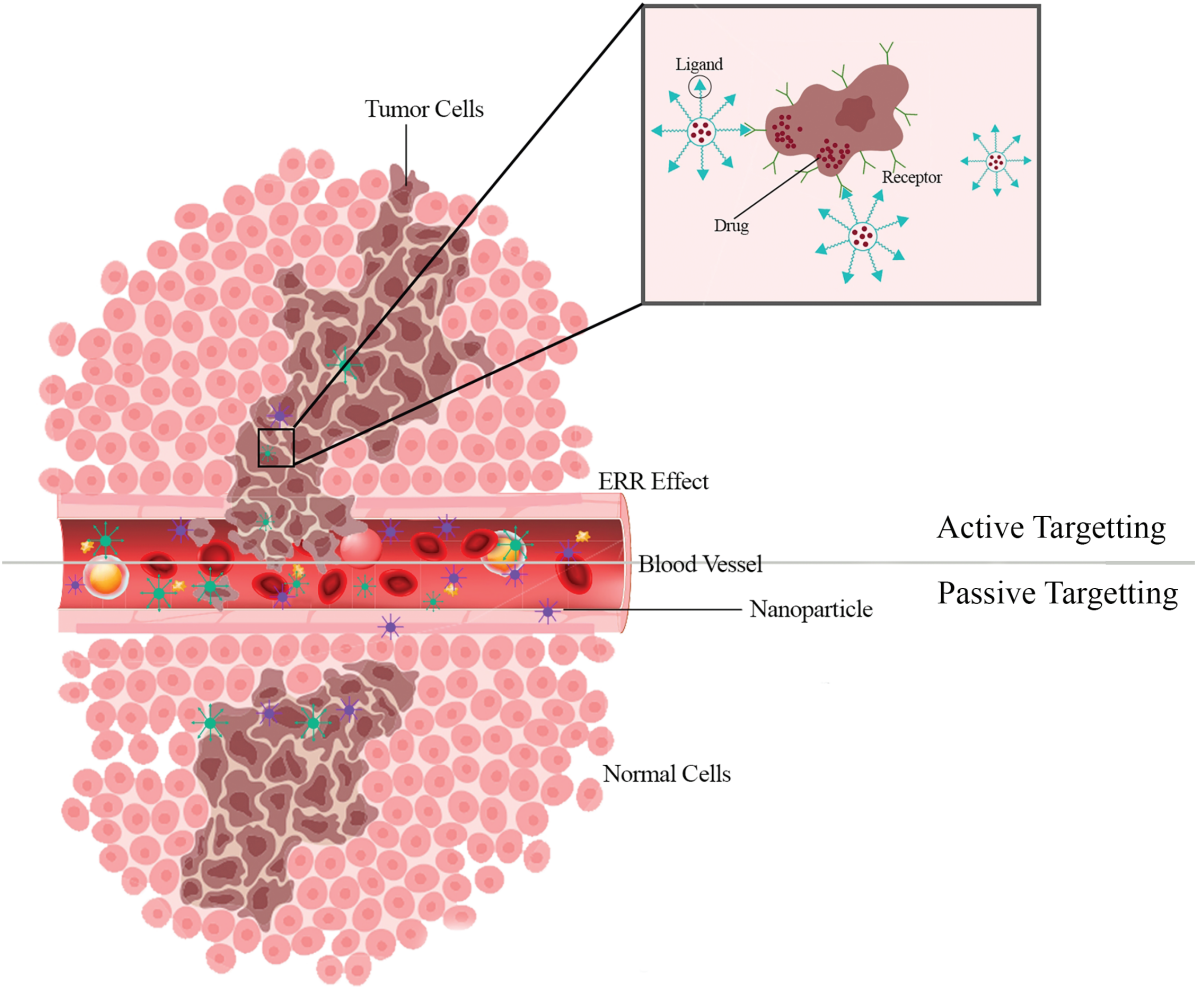
Active targeting makes it easier for tumor cells to actively take up nanoparticles. The effective localization of nanoparticles into the tumor microenvironment is made possible by passive targeting.

A tumor-targeting drug delivery system comprises a tumor recognition moiety and a cytotoxic agent coupled directly or via a linker to form a conjugate [97–100]. The fact that the endothelium of blood vessels becomes more permeable than in a healthy state under specific situations (inflammation/hypoxia, which is typical of tumors) is now well-established. Rapidly growing tumors generate new vessels or engulf existing blood vessels in response to hypoxia. These newly generated leaky vessels allow for more preferential permeation of larger macromolecules and nano-systems into the tumor stroma. Furthermore, the retention of NPs is aided by the absence of normal lymphatic outflow in the tumor [101–103]. Nanoparticle targeting can be accomplished in two ways: passive targeting and active targeting [104,105] (Fig. 3).

### Passive targeting

The size of a nanoparticle is an essential factor in determining passive targeting and biodistribution within brain tumors. Nanoparticles in the 10–100 nm size range take advantage of a brain tumor’s hyper-vascularized, leaky, and impaired lymphatic drainage system to passively target and access the intra-tumoral region while being denied access to the healthy brain tissue [106]. Passive targeting, also known as physical targeting, is based on creating a drug carrier complex resistant to removal by bodily functions such as metabolism, excretion, opsonization, and phagocytosis, allowing the complex to circulate in the bloodstream and be transmitted [107–109]. Due to physicochemical or pharmacological considerations, passive targeting installs a drug or drug-carrier structure at a specified spot. Passive targeting leverages the enhanced permeability and retention (EPR) effect, allowing nanoparticles to accumulate in brain tissue without specific targeting mechanisms. Due to the vast phenotypic variability of malignant cells and tumors, only a few regularly adopted approaches for targeting tumors and tumor cells exist. Many cancers exhibit an EPR effect that can be utilized for passive anti-tumor drug targeting [110]. The preferential accumulation of chemotherapeutic drugs in solid tumors is the most well-known example of passive targeting. As a result, tumor tissues have increased vascular permeability compared to healthy tissue. Passive targeting allows for highly selective ligand-receptor interactions, which permits precise targeting at the site of interest. On the other hand, active targeting involves functionalizing nanoparticles with ligands that bind to specific receptors on the BBB, facilitating receptor-mediated transcytosis. This process is influenced by the nanoparticle’s size, surface coating, and charge, which are optimized to improve BBB crossing efficiency. Smaller nanoparticles with a neutral or slightly negative charge and a hydrophilic surface coating are generally favored for efficient BBB penetration [111].

### Active targeting

The active targeted drug delivery system is based on a technology that delivers a specific amount of a therapeutic or diagnostic agent or both to an affected tissue/organ of the body [112,113]. Targeting ligands are added to the surface of the Nanocarrier in active targeting to bind to suitable receptors expressed at the target site. The ligand is chosen to attach to a receptor that is overexpressed by tumor cells or vessels but not by normal cells [114–116]. Targeted receptors should also be expressed uniformly on all targeted cells. Due to the “binding-site barrier,” the ligands’ binding affinity influences tumor penetration. Due to the dynamic flow environment of the bloodstream, high-affinity binding appears to be desirable for sites where cells are readily accessible, such as tumor vasculature [117]. The diverse moieties investigated thus far as targeting ligands include carbohydrates (e.g., galactose), monoclonal antibodies (e.g., anti-Her2, anti-EGFR), peptides (e.g., Arg-Gly-Asp or RGD), proteins (e.g., lectins, transferrin), vitamins (e.g., vitamin D), and aptamers (e.g., RNA aptamers against HIV glycoprotein) [118]. Some of the drawbacks of passive targeting approaches may be overcome by using a more active targeting mechanism. The discovery of disease-specific biomarkers has paved the way for this approach in the nanomedicine sector [119]. Active targeting usually entails using one or more targeting moieties attached to the nanoparticle’s surface that interact particularly with antigens or receptors that are either uniquely expressed or overexpressed on tumor cells compared to normal tissues [120]. If inter-finalizing receptors are targeted, this technique also has the added benefit of boosting nanoparticle transport into cells via a specific pathway after the particles reach the tumor extracellular space. Moreover, ligands can be used to target intravascular tumor cells or tumor blood vessels’ endothelial cells to increase nanoparticle accumulation within the disease location [121]. Because several molecular mechanisms of chemoresistance characterize Glioblastoma (GB), developing dynamically targeted NPs to surface cell markers, signaling pathways, and the tumor microenvironment poses an exciting and demanding possibility. These methods work by attaching particular ligands to the NPs’ structure, allowing for the selective identification of specific receptors overexpressed on tumor cell surfaces [122].

Solid lipid nanoparticles (SLNs) have emerged as a promising vehicle for delivering therapeutic agents across the BBB, offering a range of advantages such as controlled drug release, extended circulation within the bloodstream, precise targeting, and reduced potential for toxicity [123]. Notably, researchers have made significant strides in this field, with the development of lipid-coated mesoporous silica nanoparticles modified with Angiopep-2. These innovative nanocarriers have demonstrated the ability to transport paclitaxel (PTX) through the BBB, facilitated by the low-density lipoprotein receptor-related protein 1 (LRP1), thus showing great potential for treating gliomas [71].

## Conclusion

Nanoparticle-based therapies show great promise for the diagnosis and treatment of brain tumors, offering advantages such as high sensitivity, lower toxicity, and enhanced safety compared to traditional methods. These therapies, particularly those utilizing nanomaterials, have been developed to transport therapeutic drugs across the blood-brain barrier (BBB), a major obstacle in treating central nervous system diseases. Nanomaterials are valuable due to their high drug-loading capacity, stability, prolonged circulation in the bloodstream, controlled drug release, and targeted impact. However, existing *in vitro* and *in vivo* BBB models face limitations, which complicates the clinical translation of these nanodrug delivery systems.

Navigating the regulatory landscape for nanoparticles in medicine is challenging, with ethical considerations focused on patient safety, potential long-term effects, and managing the risks associated with nanotoxicity. It is crucial to balance the benefits of nanomedicine with the responsibility to avoid harm, adhering to ethical principles like non-maleficence and beneficence. Ongoing research and dialogue will be essential to address these challenges, ensuring that the application of nanomedicine in treating brain cancer is both safe and effective.

Financial barriers, such as high production costs and limited insurance coverage, can limit patient access to these innovative therapies. To address these challenges, efforts should be made to optimize manufacturing processes, advocate for policy changes, and explore public-private partnerships to reduce costs and improve accessibility.

Nanotechnology is becoming increasingly important in various scientific fields due to its numerous applications and benefits. The primary limitation in treating central nervous system diseases is the difficulty of drug transmission across the BBB, a challenge that nanotechnology is helping to overcome. Nanotechnology’s ability to transport drugs to targeted organs offers higher efficacy and fewer side effects on other organs. Future advancements in nanotechnology will likely lead to the development of multifunctional nanoparticles that can cross the BBB more efficiently, potentially incorporating targeting ligands or magnetic properties to direct them to tumor sites. The integration of diagnostic and therapeutic functions, known as theranostics, will enable real-time monitoring of treatment responses.

In gene therapy, nanoparticles could deliver genetic material directly to brain tumor cells to correct genetic mutations or silence oncogenes. The use of nanoparticles in immunotherapy to help the immune system recognize and attack brain tumor cells also holds significant potential. The future of nanoparticles in brain tumor treatment may involve personalized medicine, with treatments tailored to the unique molecular profile of each patient’s tumor.

This study highlights recent advancements in nanotechnology for brain cancer treatment, particularly the use of nanoparticles to cross the BBB and target brain tumors with precision. These advancements offer promising alternatives to conventional therapies, which often fail due to the restrictive nature of the BBB. Additionally, the integration of theranostic functions in these nanoparticles represents an important development in personalized medicine.

Despite these advancements, the study acknowledges several limitations. The *in vitro* and *in vivo* models used may not fully replicate the complexity of the human brain environment. Furthermore, while this study discusses the potential of various nanoparticles, it lacks comprehensive clinical data on the long-term safety and efficacy of these therapies in human patients. The regulatory and ethical challenges associated with nanomedicine, including potential nanotoxicity and the high cost of nanoparticle production, also need to be addressed.

Future research should focus on developing more accurate *in vitro* and *in vivo* models that better mimic the human BBB and brain tumor environment. Clinical trials are necessary to validate the safety and efficacy of these therapies in human patients. Additionally, optimizing the cost-effectiveness of nanoparticle production will be crucial to making these advanced therapies more accessible in clinical practice. Personalized nanomedicine has the potential to become a key approach in treating brain tumors, particularly for patients who do not respond to traditional therapies.

Finally, this study recognizes the importance of considering biological variables, such as sex and gender, which may influence the effectiveness and safety of nanoparticle-based therapies. Future research should take these factors into account to develop more personalized and effective treatments for brain cancer.

In summary, nanotechnology holds significant potential for treating brain cancer, and this study provides valuable insights into overcoming the challenges posed by the BBB. As the field of nanomedicine continues to advance, addressing current limitations and pursuing innovative solutions will be essential for improving the efficacy and accessibility of these therapies, ultimately leading to better outcomes for patients with brain cancer.

## Data Availability

Not applicable.
